# A Novel *IL3-ETV6* Fusion in Chronic Eosinophilic Leukemia Not Otherwise Specified With t(5; 12) (q31; p13): A Case Report and Literature Review

**DOI:** 10.3389/fonc.2022.887945

**Published:** 2022-06-07

**Authors:** Cenzhu Zhao, Man Wang, Yuchen Zhan, Yang Xu, Suning Chen, Qinrong Wang, Jingnan An, Tianhui Liu

**Affiliations:** ^1^ National Clinical Research Center for Hematologic Diseases, Jiangsu Institute of Hematology, The First Affiliated Hospital of Soochow University, Suzhou, China; ^2^ Institute of Blood and Marrow Transplantation, Collaborative Innovation Center of Hematology, Soochow University, Suzhou, China

**Keywords:** case report, fusion gene, t(5;12)(q31;p13), *IL3-ETV6*, CEL-NOS, eosinophilia

## Abstract

Chronic eosinophilic leukemia not otherwise specified (CEL-NOS) is classified as Myeloproliterative Neoplasms (MPN) and refers to chronic eosinophilic leukemia with some atypical recurrent genetic evidence(1). A rare fusion of *ACSL6*-*ETV6* was previously identified in patients with the t(5;12) (q31; p13) karyotype(2). Here, we report a case of CEL-NOS with a translocation of t(5;12) (q31; p13) and identify *IL3-ETV6* transcription, which has not been identified in hematologic diseases. In this patient, eosinophilia was observed. And compared with CEL-NOS patients without *ETV6* fusion, a higher mRNA expression level of *IL3* was found. After failing treatment with dasatinib, the patient was given hydroxyurea (HU). Subsequently his white blood cell (WBC) and eosinophils decreased significantly and remained in the normal range until publication. Due to the side effects, treatment with HU was replaced by PEG-interferon (PEG-IFN). What’s more, we summarized the case in our study and 21 patients with the karyotype of t(5; 12) (q31; p13) reported by other groups. It was found that most of them had similar clinical manifestations of eosinophilia and tyrosine kinase inhibitor (TKI) insensitivity. The ectopic mRNA expression of *IL3* may be the main cause of eosinophilia, and HU and prednisone acetate (PAT), as well as IFN, were considered treatments for this group.

## Introduction

Identification of specific chromosomal changes and recurrent gene translocations is crucial for the treatment and prognosis of patients with hematologic diseases, which is indispensable for the WHO classification of tumors of hematopoietic and lymphoid tissues. Recurrent *IL3-IGH* rearrangement [t(5;14)(q31.1;q32.1)] has been recognized as an entity of B-cell acute lymphoblastic leukemia, with distinct increased production of *interleukin-3 (IL3)* and subsequently characteristic reactive eosinophilia (1). It was supposed that ectopic expression of *IL3* participated in the multistep process of leukemia (2). Additionally, hematologic malignancies with eosinophilia are often associated with rearrangements of genes such as *PDGFRA* (4q12), *FILIP1-PDGFRB* (5q31-33) and *FGFR1* (8p11-12) (3). A fusion of *ACSL6*-*ETV6* with chromosome translocation of t(5;12) (q31;p13) was first reported in 1999 (4). In the study by Yahata et al. (5), patients with this genetic abnormality often have accompanying eosinophilia. In our study, a novel *IL3-ETV6* was identified in chronic eosinophilic leukemia not otherwise specified (CEL-NOS), and a significant increase in eosinophils and prominent overexpression of *IL3* mRNA were found. Moreover, we found that 22 patients with t(5;12) (q31;p13) (including 1 patient in our study and 21 patients reported by other groups) had common clinical characteristics (4–20). It was suggested that eosinophilia or hematologic malignancies with eosinophilia may have more undifferentiated subtypes.

## Case Report

**Figure 1:**
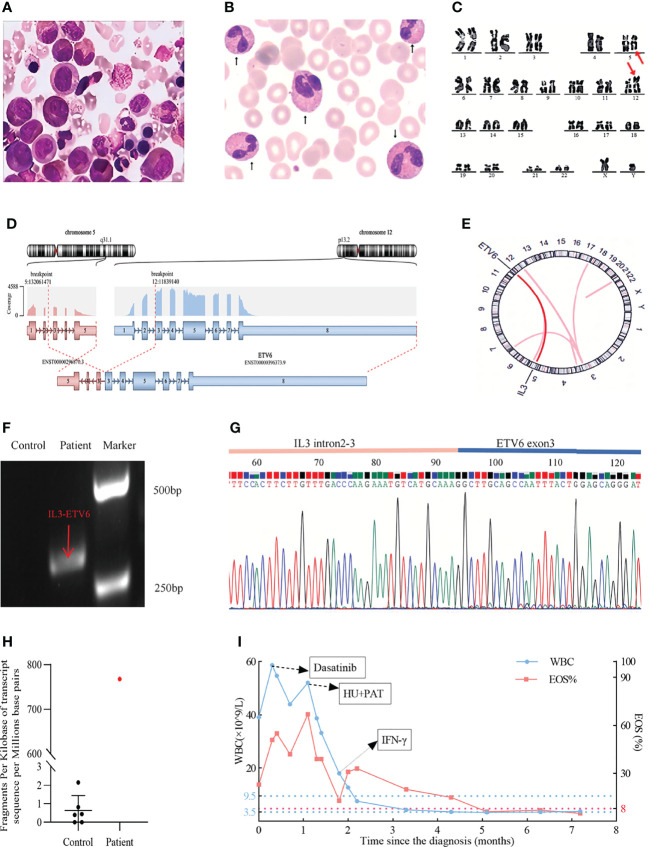
**(A)** A significantly increased eosinophil ratio was shown on a bone marrow (BM) smear (Wright–Giemsa stained, x 100). **(B)** Significantly increased eosinophilia was observed on a peripheral blood (PB) smear (× 100), the arrows delineated eosinophils. **(C)** Karyotypic analysis of BM showed the translocation of t(5; 12) (q31; p13) (delineated by arrows). **(D)** RNA-sequencing analysis indicated the *IL3-ETV6* fusion. **(E)** Circos plot displaying the interconnectivity between *IL3* and *ETV6*. **(F)** Amplified *IL3-ETV6* transcripts by RT–PCR. Control: water. The predicted product was 300bp, and the patient’s PCR product was indicated by red arrow. **(G)** Sanger sequencing of the PCR product (*IL3-ETV6*). **(H)** *IL3* mRNA was obviously more highly expressed than in other eosinophilia patients with normal karyotypes. Control: CEL-NOS patients with normal karyotype, FPKM: fragments per kilobase of transcript sequence per millions base pairs sequenced. **(I)** Fluctuation of the patient’s peripheral white blood cell count (WBC, ×10^9^/L) and eosinophil ratio (EOS, %) during treatment.

A 38-year-old man was admitted due to elevated levels of white blood cell (WBC) and eosinophils accompanied by splenomegaly on June 4, 2021. He had no history of radiation or drug exposure. The complete blood count and main biochemistry examinations were shown in **Table 1**. Bone marrow (BM) and peripheral blood (PB) smears showed a high ratio of eosinophils (26% in BM, 47% in PB) (**Figures 1A, B**), and the ratio of granulocytes: erythrocytes in BM was 13.3:1 (1.28-5.95:1). The percentage of myeloblasts in peripheral blood and bone marrow was 1%, respectively. Additionally, BM biopsy showed an increased number of myeloblasts dominated by eosinophils (>80%). The karyotype analysis revealed 46, XY, t(5;12) (q31; p13) (**Figure 1C**). *BCORL1* (p.S803fs, VAF 19.26%), *RUNX1* (p.S291fs, VAF36.05%), *KMT2C* (p.R894Q, VAF 5.69%), *CCND1* (p.276_276del, VAF 5.11%), *PTPN1* (1p.Y375C, VAF 27.38%), *STAT5B* (p.Q368fs, VAF 5.11%) mutations (**Supplementary Table 1**) were detected by next-generation sequencing covering 161 genes reportedly mutated in hematologic malignancies (**Supplementary Table 2**). Moreover, the nested polymerase chain reaction (PCR) for *BCR-ABL1*, *FIP1L1-PDGFRα*, *ETV6-PDGFRα*, and *ETV6*-*PDGFRβ* fusion genes, which are commonly detected in eosinophilia or hematologic malignancies with eosinophilia, were all negative. Supplementary medical history showed that the patient had skin itching and rash for more than 10 years.

**Table 1:** The CBC and main biochemistry examniations of this patient.

Projects	Normal range	Counts
Hb	120-160 g/L	120
WBC	3.5-9.5 ×10^9^/L	39.1↑
eosinophil	0.02-0.52 × 10^9^/L	8.8↑
basophil	0.00-0.06 × 10^9^/L	0.3↑
PLT	125-350 × 10^9^/L	179
Fer	23-336ng/ml	417↑
VitB12	180–914 pg/mL	>1500↑
EPO	4.3–29 MIU/mL	32.4↑
LDH	<248 U/L	196
IgE	0-100 IU/mL	4.7

CBC, complete blood count; Hb, hemoglobin; WBC, white blood cell; PLT, platelet; Fer, ferritin; VitB12, vitamin b12; EPO, erythropoietin; LDH, lactate dehydrogenase. ↑, Increased.

For further examination of the molecular abnormality, RNA sequencing using HiSeq (Illumina Inc, San Diego, CA, USA) was performed, which led to the identification of a novel fused mRNA, *IL3-ETV6* (**Figures 1D, E**). In this translocation, exons 3 to 5 of IL3 on chromosome 5 were spliced, inverted and fused with exons 3 to 8 of *ETV6* on chromosome 12. In addition, a transcript of *GATA2-SOCS2* (exon 1 of *GATA2* (5’UTR) fusing with exon 2 to 3 of *SOCS2* (5’UTR)) was also detected (**Supplementary Figure 1**). Reverse transcription PCR (RT–PCR) was performed to confirm the *IL3-ETV6* fusion by the following primers: forward (at *IL3* intron 2-3), 5’AAATCA CAGAGACCCCAGC3’ and reverse (at *ETV6* exon 3), 5’AAGG AGTTCATAGAGCACATCA3’, and a product of approximately 300 bp was observed as we predicted (**Figure 1F**). Sanger sequencing analysis of this product showed the detailed breakpoints and fusion segments (**Figure 1G**). The raw data can be downloaded in the **Supplement 1**. In addition, ectopic high-level expression of *IL3* mRNA was also observed in patients with *ACSL6*-*ETV6* (8, 11). Therefore, we reanalyzed the RNA-sequencing data. Compared with other CEL-NOS patients with normal karyotype, significantly higher (more than 700-fold change) fragments per kilobase of transcript sequence per million base pairs of *IL3* mRNA in this patient was found (**Figure 1H**). Meanwhile, high mRNA expression levels of *GATA2* and *SOCS2* (more than 40-fold change and 30-fold change respectively) were also observed (**Supplementary Figure 2**).

The dynamic changes in WBC and eosinophil in this patient during treatment are shown (**Figure 1I**). Owing to the success of tyrosine kinase inhibitor (TKI) in CEL-NOS reported in previous cases, the patient was first treated with dasatinib 250 mg/day for 20 days, but the high WBC and eosinophil counts were maintained. Then, treatments with hydroxyurea (HU) 150 mg/day and prednisone acetate (PAT) 30 mg/day for 20 days were given, and the WBC count decreased from 38.7×10^9^/L to 18×10^9^/L, and eosinophil count decreased from 15.2×10^9^/L to 2.29×10^9^/L. However, due to the fever and gastrointestinal discomfort associated with HU, PEG-interferon (PEG-IFN) was used as an alternative. His WBC and eosinophil counts in peripheral blood soon returned to the normal range. The patient was then followed up for more than seven months, and the WBC and eosinophil counts remained stable by the time of this report.

Due to the rare incidence and limited description, we summarized the case in our study and 21 patients with the karyotype of t(5; 12) (q31; p13) reported by other groups from 1988 to 2021 (4–20) (**Table 2**). According to these reports, 19 cases were classified as MDS/MPD by the 2016 WHO criteria (21), 2 were AML and 1 was T-ALL. Most of these patients were male (17/22), and most of them had accompanying eosinophilia (normal range 0.4%-8%) (14/18). Unlike TKI treatment, which failed in these patients (4/4), HU with/without PAT or PEG-IFN treatment hydroxyurea or interferon treatment led to hematologic and cytogenetic remissions (12/16).

**Table 2:** Clinical features of 21 patients with t(5; 12) (q31; p13) with cases reported in the literature and the 1 patient in our center.

Patient N0.	Age(years)/sex	Diagnosis	WBC(10^9^/L)	EO(%)	Hb(g/L)	Platlet(10^9^/L)	Fusion gene	Treatment	Disease Status	Follow-up	Reference
1	30/M	AML(relapse)	41.1	42	134	370	uncertain,ETV6-PDGFRβ?	chemotherapy	Relapsed	1,-,dead of sepsis	(5)
2	41/M	CMMoL	32.4	11	152	202	NM	HU	Remission	31+	(15)
3	59/M	EoL	20.9	10	42	40	NM	BU,6-MP	NM	3-,dead with uncontrolled disease?	(19)
4	4/M	T-ALL relapse	214	NM	94	108	NM	polychemotherapy	Relapsed	12,-,dead of ARDS	(7)
5	44/M	MDS	27.5	29	45	73	ETV6-ACSL6	TKI, Hu, HSCT	Remission	24+	(8)
6	52/M	CEL	NM	NM	NM	NM	ETV6-ACSL6	TKI, HU	Remission	27+	(9)
7	67/F	aCML-AP	79	NM	77	55	NM	HU	Remission	6,-,dead of disease progression	(20)
8	16/F	CEL	48	85	69	61	NM	Hu+PAT,IFN,TKI,splenectomy, polychemotherapy	NR	22,-,dead of disease progression	(10)
9	49/M	aCML	NM	↑	NM	NM	ETV6-ACSL6	HU; HU+IFN	NR	6,-,dead	(11)
10	53/M	ph(-)CML--AML-M5	32.0	*3*	57	156	NM	BU,polychemotherapy	NM	9,-,dead of sepsis	(12)
11	40/M	ph(-)CML	46.9	12	68	52	NM	allo-HSCT	NR	14,-,dead of disease progression	(12)
12	43/M	HES	26.5	18	NM	NM	NM	HU,VCR,allo-HSCT	Remission	48,-,dead of infection	(13)
13	68/F	MDS	8.8	3	84	37.5	ETV6-ACSL6	AraC	Remission	uncertain-,dead of sepsis	(4)
14	27/M	MDS	41.1	42	NM	NM	ETV6-ACSL6	HU,AraC,VCR,IFN	Relapsed	1,-,dead of sepsis	(4)
15	53/M	AML	59.5	69	NM	59.5	ETV6-ACSL6	polychemotherapy	Relapsed	9,-,dead of eos infiltration	(4)
16	16//M	aCML	46.4	6	120	213	NM	IFN.HU	Remission	NM	(14)
17	8/F	MDS	33.7	6.2	129	322	NM	HU	Remission	84+	(16)
18	7/M	MDS	NM .	↑	NM	NM	NM	HU	Remission	48+	(17)
19	59/M	EoL	136	69	125	116	NM	HU,BU	Remission	3,-,dead of cerebral infarction	(18)
20	29/M	PV	11.2	16	210	522	ETV6-ACSL6	HU	Remission	42+	(6)
21	31/F	PV/AML	18.2	NM	130	*278*	ETV6-ACSL6	HU,polychemotherapy	Relapsed	11,-,dead of hemorrhage	(6)
22*	38/M	CEL-NOS	39.1	22.6	120	60	IL3-ETV6	TKI/Hu+PAT/IFN-γ	Remission	7+	–

F, female; M, male; AML, acute myelocytic leukemia; CMMoL, chronic myelomonocytic leukemia; EoL, eosinophilic leukemia; T-ALL, T-cell acute lymphoblastic leukemia; MDS, myelodysplastic syndrome; aCML-AP, atypical chronic myeloid leukemia, acceleration phase; CEL, chronic eosinophilic leukemia; HES, hypereosinophilic syndrome; PV, polycythemia vera; Eo%, percentage of eosinophils (normal range, 0.04-8%); NM, not mentioned; allo-HSCT, allogenic hematopoietic stem cell transplantation; IFN, interferon; HU, hydroxyurea; PAT, prednisone acetate; TKI, tyrosine kinase inhibitor; Ara, cytarabine, BU, busulfan; VCR, vincristine; NR, non-remission; +, alive; -, dead; ?,not sure; ↑, increased; *, patient in our study.

## Discussion

CEL-NOS is a rare disease with eosinophilia and nonspecific clonal cytogenetic abnormalities. Patients diagnosed with CEL-NOS have the clinical manifestations of increased eosinophils and organ damage, with the risk of transformation into hematologic malignancies (3). However, the genetic abnormality of this disease has not been fully delineated, which will be essential for precise diagnosis and treatment.

Due to the rare incidence and limited description, we summarized all the reported cases with the karyotype of t(5; 12) (q31; p13). A fusion of *ACSL6-ETV6* was previously identified in several patients with t(5;12) (q31; p13) (8, 9, 12, 14, 20). *ACSL6*, also named as *ACS2*, is considered as a suppressor gene in leukemia and involved in the metabolic process of leukemia cells (22). *ETV6* on chromosome 12 plays a pivotal role in the regulation of myeloid hematopoiesis (23). It encodes a transcriptional repressor that plays a critical role in hematopoiesis and maintains HSCs. It is believed that the rearrangement of *ETV6* and frequent loss of *ETV6* expression could be genetic events that induce leukemia (24).

In our study, we did not observe ACSL6-ETV6 but a novel IL3-ETV6 transcript. 5q31-33 is a common fragile fragment that includes tissue-derived growth factor receptor B (PDGFRB), acyl-CoA synthetase long chain family member 6 (ACSL6), and interleukin 3 (IL3). In the chromatin 5q31, IL3 was adjacent to ACSL6 by 50KB. ACSL6-ETV6 was analyzed by FISH probes (a resolution at least 100kB-150KB) in previous reports (4, 6, 9, 11). Therefore, IL3 and ACSL6 could not be distinguished by FISH analysis, and we suspected that some patients expressing IL3-ETV6 were ignored due to technology limitations.

According to our description, the *IL3* (exon 3-5) was reversely fused to *ETV6* (exon 3-8), which resulted in the loss of the start codon for both genes. Out-of-frame fusions such as *ACSL6-ETV6*, *IL3-IGH* may be involved in pathogenesis was reported previously (1, 11). In all these cases, abnormally high mRNA expression of *IL3* might be the molecular characteristic of t(5;12) (q31; p13). In our study, we found high expression level of *IL3* mRNA by RNA sequencing. *IL3* can promote proliferation and differentiation of eosinophils (11). Additionally, CD123, an *IL3* receptor, was hypothesized to induce JAK-STAT-dependent cell survival and proliferation in an autocrine manner (25). *IL3* rearrangement not only promotes the proliferation of eosinophils but also influences malignant blasts (1, 25). Thus, we speculated that high expression of *IL3* may be a consequence of *IL3-ETV6* fusion and may play a role in the pathogenesis. But it needs to be further confirmed.

In this case, exon 1 of *GATA2* (5’UTR) fused with exon 2 of *SOCS2* (5’UTR). We also found there were high mRNA expression levels of *GATA2* and *SOCS2*. However, there were few literatures on the functions of *GATA2* or *SOCS2* in eosinophilia. We cannot find more evidence for the correlation of *GATA2-SOCS2* with this disease. In summary, we considered that *IL3-ETV6* is more likely to cause the progression of eosinophilia but not *GATA2-SOCS2*.

Similar clinical characteristics of eosinophilia and high mRNA expression levels of *IL3* were found in *IL3-ETV6* and *ACSL6-ETV6* cases (8, 11). Thus, we believe that eosinophilia associated with IL3 high expression could be treated in a similar manner.

Although most patients with the karyotype of t(5; 12) (q31; p13) responded well to hydroxyurea and interferon, there were still risks of hematologic malignant progression and recurrence. Therefore, long-term follow-up should be conducted for this group. Some patients can achieve remission after chemotherapy or transplantation (13, 20). Allogeneic transplantation and intensive chemotherapy should be considered for patients with malignant transformation. In addition, JAK2 or CD123 might be a therapeutic target for some *IL3* mRNA overexpressing and eosinophilia patients (25, 26). In study of Pellier et al. (16), an *IL5* monoclonal antibody also effectively inhibited the proliferation of eosinophils. Recently, an adeno-associated virus (AAV) coding for an anti-eosinophil monoclonal antibody was shown to persistently suppress eosinophil numbers in blood, thus reducing eosinophil tissue invasion and organ dysfunction in a murine model of CEL-NOS (27). However, targeted therapy experience for patients with the karyotype of t(5; 12) (q31; p13), especially people with atypical recurrent molecular abnormalities such as *IL3-ETV6*, is still lacking.

In conclusion, we reported a novel *IL3-ETV6* fusion in CEL-NOS with a high level of IL3 mRNA expression. RNA sequencing is valuable to identify some occult genetic abnormalities in eosinophilia and to help further understand the disease. By summarizing the previous literatures, the similar clinical characteristics and unfavorable outcomes of these patients with t(5; 12) (q31; p13) were found, which may indicate a novel subtype of hematologic malignancy.

## Data Availability Statement

The datasets presented in this study can be found in online repositories. The names of the repository/repositories and accession number(s) can be found below: https://www.ncbi.nlm.nih.gov/bioproject/?term=PRJNA812744.

## Ethics Statement

The studies involving human participants were reviewed and approved by the First Affiliated Hospital of Soochow University. The patients/participants provided their written informed consent to participate in this study. Written informed consent was obtained from the individual(s) for the publication of any potentially identifiable images or data included in this article.

## Author Contributions

Contribution: TL and JA were the principal investigators. MW, CZ, and YZ performed most of the experiments. SC, QW and YX performed clinical analysis. CZ, TL, and JA wrote the manuscript. All authors contributed to the article and approved the submitted version.

## Funding

This work was supported by grants from the Excellent Youth Science Fund of Jiangsu Province (BK20211553), the Natural Science Foundation of China (81700139, 82070187, 81870120 and 82000157), the Key R&D Program of Jiangsu Province (BE2019655), the Translational Research Grant of NCRCH (2021ZKMB01) and the Natural Science Fund of Jiangsu Province (BK20170360, BK20190173).

## Conflict of Interest

The authors declare that the research was conducted in the absence of any commercial or financial relationships that could be construed as a potential conflict of interest.

## Publisher’s Note

All claims expressed in this article are solely those of the authors and do not necessarily represent those of their affiliated organizations, or those of the publisher, the editors and the reviewers. Any product that may be evaluated in this article, or claim that may be made by its manufacturer, is not guaranteed or endorsed by the publisher.

## Supplementary Material

Supplement 1 Raw sequencing data from patient’s RNA showed the *IL3-ETV6* fusion.
