# Supplementary material for: A Novel IL3-ETV6 Fusion in Chronic Eosinophilic Leukemia Not Otherwise Specified With t(5; 12) (q31; p13): A Case Report and Literature Review
Source: Front Oncol. 2022 Jun 7;12:887945. doi: 10.3389/fonc.2022.887945 (PMC9213071; doi:10.3389/fonc.2022.887945)
Supplement: Supplement 1 — Raw sequencing data from patient’s RNA showed the IL3-ETV6 fusion. [file DataSheet_1.pdf]

Supplementary Information

A novel *IL3-ETV6* fusion in chronic eosinophilic leukemia not otherwise specified with t(5; 12) (q31; p13): A case report and literature review

Cenzhu Zhao[1*], Man Wang[1*], Yuchen Zhan[1*], YangXu[1,2], Suning Chen[1,2], Qinrong Wang[1#], Jingnan An[1,2#], Tianhui Liu[1,2#]

contents

Supplementary Figure Legends
Supplementary Figures
Supplementary Tables

Supplementary Figure Legends

Supplementary Figure 1. RNA-sequencing analysis revealed the fusion of *GATA2-SOCS2*. (A) The diagram of *GATA2-SOCS2*. **(B)** The circos plot indicated *GATA2-SOCS2*.

Supplementary Figure 2. The RNA sequencing data of *GATA2* and *SOCS2* in this case and other CEL-NOS patients with normal karyotype. (A) *GATA2* mRNA expression. **(B)** *SOCS2* mRNA expression. Note: Control: CEL-NOS patients with normal karyotype, FPKM: fragments per kilobase of transcript sequence per millions base pairs sequenced.

Supplementary Figure 1.

(A) (B)

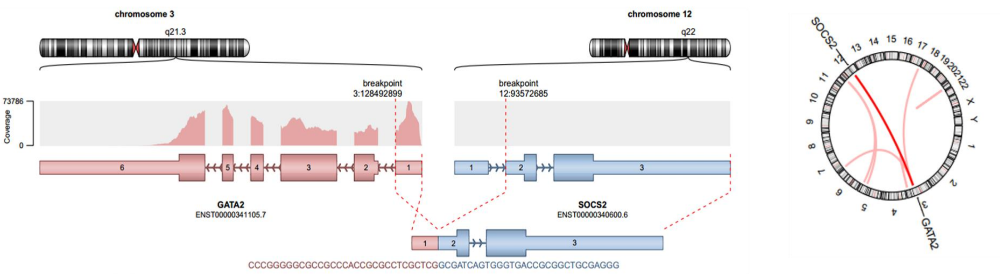

Supplementary Figure 2.

(A) (B)

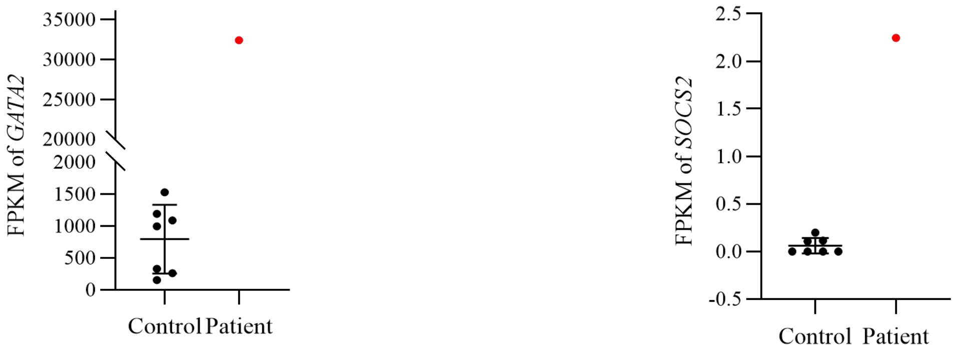

Supplementary Table 1. Summary of mutations presented in this case.

Mutation Genes	Name of Mutation	Mutation frequency (sequencing depth)
BCORL1	NM_001184772:exon3:c.2407_2410del:p.S803fs	19.26% (405X)
RUNX1	NM_001001890:exon5:c.871dupT:p.S291fs	36.05% (921X)
KMT2C	NM_170606:exon16:c.G2681A:p.R894Q	5.69%(439X)
CCND1	NM_053056:exon5:c.826_828del:p.276_276del	5.11%(1291X)
PTPN11	NM_001330437:exon10:c.A1124G:p.Y375C	27.38%(409X)
STAT5B	NM_012448:exon9:c.1102delC:p.Q368fs	5.11%(1448X)

Note: At 200 sequencing depth, the sensitivity was about 10% (mutation frequency could be reported as 10% or above), and at 500 sequencing depth, the sensitivity was about 5% (mutation frequency could be reported as 5% or above).

Supplementary Table 2. The panel of 161 gene mutations.

Genes	Detected Region	Genes	Detected Region	Genes	Detected Region	Genes	Detected Region
ABL1	CDS	***DDX3X***	CDS	***MAP2K1***	exon 2-3	***RPL10***	CDS
ANKRD26	Exon 1/ 5'UTR	***DDX41***	CDS	***MAPK1***	CDS	***RPL5***	CDS
ARID1A	CDS	***DHX15***	CDS	***MAX***	CDS	***RPS14***	CDS
ASXL1	CDS	***DIS3***	CDS	***MED12***	CDS	***RUNX1***	CDS
ASXL2	CDS	***DNM2***	Exon 8/13/16/18/20	***MEF2B***	Exon 2-3	***SETBP1***	Exon 4
ATG2B	CDS	***DNMT3A***	CDS	***MPL***	Exon 10	***SETD2***	CDS
ATM	CDS	***DNMT3B***	CDS	***MYC***	CDS	***SF1***	CDS
B2M	CDS	***EED***	CDS	***MYD88***	CDS	***SF3A1***	CDS
BCL2	Exon 2	***EGR1***	CDS	***NF1***	CDS	***SF3B1***	Exon 12-15
BCL6	5'UTR	***EP300***	CDS	***NFkB***	CDS	***SMC1A***	CDS
BCOR	CDS	***ETNK1***	Exon 3	***NOTCH1***	CDS	***SMC3***	CDS
BCORL1	CDS	***ETV6***	CDS	***NOTCH2***	CDS	***SPEN***	Exon 11
BIRC3	CDS	***EZH2***	CDS	***NOTCH3***	CDS	***SRP72***	CDS
BRAF	CDS	***FAM46C***	CDS	***NOTCH4***	CDS	***SRSF2***	CDS
BRINP3	CDS	***FAT1***	CDS	***NPM1***	Exon 11	***STAG2***	CDS
BTK	Exon 5/11/14-19	***FBXW7***	CDS	***NRAS***	Exon 2-3	***STAT3***	CDS
CALR	Exon 9	***FGFR1***	CDS	***NT5C2***	Exon 9-16	***STAT5A***	CDS
CARD11	CDS	***FGFR3***	CDS	***P63***	CDS	***STAT5B***	CDS
CASP8	Exon 10	***FLT3***	Exon 14/15/20	***PAX5***	CDS	***SUZ12***	CDS
CBL	Exon 8-9	***FOXO1***	CDS	***PDGFRA***	CDS	***TAL1***	Exon 3
CCND1	CDS	***GATA1***	CDS	***PDGFRB***	CDS	***TCF3***	Exon 6/15/17
CCND2	Exon4-5	***GATA2***	CDS	***PHF6***	CDS	***TERT***	CDS
CCND3	CDS	***GATA3***	CDS	***PIGA***	CDS	***TET1***	CDS
CCR4	CDS	***GNA13***	CDS	***PIM1***	CDS	***TET2***	CDS
CD28	CDS	***ID3***	CDS	***PLCG1***	CDS	***TNFAIP3***	CDS
CD58	Exon 2-3	***IDH1***	Exon 4	***PLCG2***	CDS	***TNFRSF14***	Exon 1-6
CD79A	CDS	***IDH2***	Exon 4	***PML***	Exon 1-6	***TP53***	CDS
CD79B	CDS	***IKZF1***	CDS	***PPM1D***	CDS	***TPMT***	CDS
CDC25C	Exon 8	***IL7R***	CDS	***PRDM1***	CDS	***TRAF3***	CDS
CDKN1B	CDS	***IRF4***	CDS	***PRF1***	CDS	***U2AF1***	CDS
CDKN2A	CDS	***JAK1***	CDS	***PRKCB***	CDS	***USP7***	CDS
CDKN2B	CDS	***JAK2***	Exon 12-16/20-21	***PRPF8***	CDS	***WHSC1***	CDS
CEBPA	CDS	***JAK3***	CDS	***PRPS1***	CDS	***WT1***	CDS
CNOT3	Exon2-5	***KDM6A***	CDS	***PTEN***	CDS	***XPO1***	CDS
CREBBP	CDS	***KIT***	Exon 2/8-11/13/17	***PTPN11***	CDS	***ZBTB7A***	CDS
CRLF2	Exon 6	***KLF2***	Exon 1-3	***RAD21***	CDS	***ZMYM3***	CDS
CSF3R	CDS	***KMT2A***	CDS	***RARA***	Exon 5-7	***ZNF384***	CDS
CSMD1	CDS	***KMT2C***	CDS	***RB1***	CDS	***ZRSR2***	CDS
CSNK1A1	Exon 2-4	***KMT2D***	CDS	***RBBP6***	CDS		
CUX1	CDS	***KRAS***	Exon 2-4	***RELN***	CDS		
CXCR4	CDS	***SH2B3***	CDS	***RHOA***	CDS		

Note: Next-generation sequencing was used for the detection of assigned regions listed in the form.
Abbreviation: CDS, coding sequence; 5'UTR, 5' Untranslated Regions.
